# Targeting the Adenosinergic Axis in Chronic Lymphocytic Leukemia: A Way to Disrupt the Tumor Niche?

**DOI:** 10.3390/ijms19041167

**Published:** 2018-04-12

**Authors:** Tiziana Vaisitti, Francesca Arruga, Silvia Deaglio

**Affiliations:** Department of Medical Sciences, University of Turin School of Medicine & Italian Institute for Genomic Medicine (IIGM), via Nizza, 52, 10126 Torino, Italy; tiziana.vaisitti@unito.it (T.V.); francesca.arruga@iigm.it (F.A.)

**Keywords:** adenosine, ATP, chronic lymphocytic leukemia, tumor niche, immunosuppression

## Abstract

Targeting adenosine triphosphate (ATP) metabolism and adenosinergic signaling in cancer is gaining momentum, as increasing evidence is showing their relevance in tumor immunology and biology. Chronic lymphocytic leukemia (CLL) results from the expansion of a population of mature B cells that progressively occupies the bone marrow (BM), the blood, and peripheral lymphoid organs. Notwithstanding significant progress in the treatment of these patients, the cure remains an unmet clinical need, suggesting that novel drugs or drug combinations are needed. A unique feature of CLL is its reliance on micro-environmental signals for proliferation and cell survival. We and others have shown that the lymphoid niche, an area of intense interactions between leukemic and bystander non-tumor cells, is a typically hypoxic environment. Here adenosine is generated by leukemic cells, as well as by cells of myeloid origin, acting through autocrine and paracrine mechanisms, ultimately affecting tumor growth, limiting drug responses, and skewing the immune cells towards a tolerant phenotype. Hence, understanding the mechanisms through which this complex network of enzymes, receptors, and metabolites functions in CLL, will pave the way to the use of pharmacological agents targeting the system, which, in combination with drugs targeting leukemic cells, may get us one step closer to curing these patients.

## 1. Introduction

Over the past years, several findings brought adenosine triphosphate (ATP) metabolism and purinergic signaling under the spotlight of research because of their high relevance in immunology and cancer biology. While the regulation of ATP/adenosine (ADO) homeostasis is now well defined, with the functional characterization of the players involved, knowledge on the physio-pathological role of the system is still incomplete. The current view in immunology is that ATP and ADO represent a “Yin and Yang”: while ATP boosts the immune system acting as a “find-me” signal that induces the recruitment of inflammatory cells, ADO blunts these responses. For this reason, ATP is considered a danger signal that activates inflammatory responses and that is counteracted by ADO, which provides a powerful stop signal, preventing excessive tissue damage [1]. Seen in the context of cancer immunology, ADO becomes a potent immunosuppressive agent that favors cancer expansion through the creation of an immune-tolerant and tumor-supportive environment. Emerging data indicate that the purinergic and adenosinergic systems are not only critical in shaping the tumor microenvironment in solid tumors, but also in hematological malignancies, including chronic lymphocytic leukemia (CLL) [2,3].

With an incidence rate of four to six cases per 100,000 individuals per year (Available online: www.seer.cancer.gov), CLL is the most frequent leukemia in Europe and North America. The finding of accumulated mature B lymphocytes in the peripheral blood (PB) led to propose CLL simply as a disease of failed apoptosis [4]. However, CLL is now viewed as a compartmentalized leukemia with a substantial proliferative core residing in the lymph nodes (LN) [5]. There leukemic cells find the ideal soil, made of the antigen and a cocktail of accessory signals that drive B cell proliferation [6,7]. From the clinical point of view, it is easy to diagnose CLL, but difficult to estimate its prognosis. Indeed, the disease is characterized by a variable clinical course, with patients presenting indolent asymptomatic lymphocytosis alongside patients with an aggressive form of the disease associated to immunosuppression and poor responses to therapy. This observation incited a long quest for disease markers, which led to the identification of several prognosticators, which have increased our understanding of the pathogenesis of the disease. Among them, chromosomal abnormalities (e.g., deletion 17p and 11q) and mutations in different genes (e.g., *TP53*, *NOTCH1*, *SF3B1*) carry prognostic significance and are associated to a shorter time to progression and a lower overall survival [8,9,10]. Importantly, none of these aberrations seem to be a disease-driver, although they commonly insist mostly on the deregulation of cell survival and recirculation [11,12].

Novel therapeutic approaches, mainly represented by kinase inhibitors, used alone or in combination with chemo-immunotherapy are now approved for clinical use. However, results so far indicate that while they are very effective in controlling the disease, they do not cure it, arguing in favor of combination strategies [13]. Therefore, a dual approach concomitantly targeting leukemic cells while relieving immunosuppression could represent a winning strategy [14]. In this context, drugs that target the adenosinergic signaling pathway could find therapeutic applications as adjunctive tools.

This review will navigate the main aspects of the ATP-ADO axis, mainly focusing on CLL. The key features of this system will be explored, looking at expression and functions of the main players, along with novel pharmacological strategies that may carry therapeutic relevance.

### ATP/ADO Balance: Shifting from Immune-Activating to Immune-Suppressive Signals

**Figure 1:**
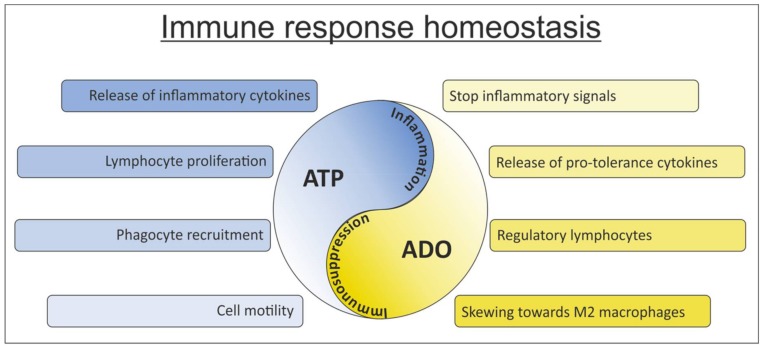
Targeting adenosine triphosphate (ATP) and adenosine (ADO): A Yin and Yang of the immune system. ATP and ADO are present in tissues or in the tumor niche at variable concentrations. Shifting the balance between these two metabolites results in inflammation (high ATP concentration) or immunosuppression (high ADO concentration). Both these extremes represent a potentially harmful condition, considering the biological consequences exerted on cells of the immune system.

In healthy tissues, ATP is mainly present inside the cells, where it reaches the millimolar range, whereas in the extracellular space it is found at low nanomolar concentration [15]. However, extracellular ATP levels can rise sharply in conditions of cellular stress or damage [16], acting as a beckon signal able to recruit phagocytes to inflammatory sites, promoting the clearance of damaged cells [17]. Physiologically, it can be degraded to ADO, which counteracts ATP signals thus representing a potent immunosuppressor [18] (Figure 1). Outside the cells, ATP is enzymatically converted to adenosine diphosphate and adenosine monophosphate (ADP and AMP, respectively) by ectonucleotidases belonging to three different families: alkaline phosphatases, ectonucleoside triphosphate diphosphohydrolases (ENTPDases, including CD39/NTPDase-1), and ectonucleotide pyrophosphatases/phosphodiesterases (ENPPs). Extracellular AMP is primarily converted to ADO by CD73 (also known as 5′-NT), a glycosyl- phosphatidylinositol (GPI)-linked membrane-bound glycoprotein [19,20,21].

ADO signaling is controlled at least in part by the enzyme adenosine deaminase (ADA), which converts it to inosine. ADA activity is strongly dependent on its interaction with the cell surface serine protease dipeptidyl peptidase IV/CD26, which acts as an ADA-binding protein. Alternatively, accumulated extracellular ADO can mediate its regulatory functions by binding to one of four type 1 (P1) ADO receptors, namely A1, A2A, A2B, and A3 [22].

All P1 receptors are G-protein-coupled, linked to calcium mobilization from intracellular stores or cyclic-AMP (cAMP) increase. Signaling via cAMP is typically associated with significant immunosuppression [23,24], while inhibition of cAMP generation after P1 engagement is generally viewed as an immune-stimulating mechanism [25]. All ADO receptors couple to mitogen-activated protein kinase (MAPK) pathways [26]. Each receptor exhibits different affinities for ADO. A1, A2A, and A3 respond to low levels of ADO (250–700 nM), representing high-affinity receptors. On the contrary, A2B is a low affinity receptor which activation occurs only under pathological conditions, where ADO accumulates at high concentration (25 μM) [27].

ATP can be released in the extracellular space via different mechanisms, including plasma membrane channel and lysis. Independently of the mechanism used, the final functional effects are a consequence of the target cell and of the receptors that ATP binds to. ATP receptors, also known as nucleotide receptors P2, comprise two subfamilies: the metabotropic P2YRs (eight members) that are coupled via G-proteins to calcium (Ca^2+^) mobilization, cAMP generation and ERK/MAPK pathway activation [28], and the ionotropic P2XRs (seven members). The latter are homo/heterotrimeric ion channels that mediate transmembrane fluxes of Na^+^, K^+^, and Ca^2+^ ions [29,30]. The main signaling mechanism used by these receptors is the alteration of intracellular ion concentration. Virtually all cell types express one or more purinergic receptors [31], including tumor cells [32].

The existence of 15 different P2 receptor subtypes, each characterized by different selectivity/affinity for the substrates and ability to form heteromeric structures, confers to purinergic signaling an exclusive plasticity, allowing for rapid modulation of cellular functions in response to local changes in nucleotide concentrations. Depending on the local nucleotide concentration changes, they can mediate chemotaxis or retention of immune cells to inflammation/tumor sites, as well as cytokine release, proliferation, and cytotoxicity [33].

## 2. The Purinergic/Adenosinergic Systems in the Tumor Microenvironment

The view of the adenosinergic network as a fine-tuned system together with its physiological impact has gained increasing relevance also in the neoplastic context [34,35,36]. The solid tumor microenvironment, as well as the lymphoid niche, are dynamic areas where cancer cells, immune cells, fibroblasts, cytokines, growth factors and the extracellular matrix mutually interact, resulting in tumor growth and progression, besides modification of bystander cells [37]. The environment surrounding tumor cells is characterized by low oxygen tension [38], a condition that induces extracellular accumulation of ATP and subsequently ADO in tumors. ATP may also accumulate in cancer milieu as consequence of spontaneous release, tumor necrosis or chemotherapy, in turn modulating the cross-talk among cancer and surrounding tissues.

Whether ATP/ADO accumulation in the extracellular milieu will prove beneficial or detrimental for the host will depend on (i) the concentration of ATP as a result of release from cells; (ii) the rate of degradation of ATP to ADO by ectonucleotidases and (iii) the panel of receptors expressed by tumor and infiltrating inflammatory cells. CD39 and CD73, both reported to be overexpressed in several tumors [32], contribute to the shaping of the tumor microenvironment [39]. The catabolic activity of CD73 is critical in generating an immunosuppressive and pro-angiogenic ADO “halo” that contributes to cancer progression [16,40,41,42]. Even if purinergic receptors have emerged as central players in tumor development, invasion and progression, acting not only on immune-infiltrating cells but also on cancer cells, their role is somewhat controversial [43]. Activation or inhibition of selected P2 receptor subtypes can indeed bring about tumor cell death/growth inhibition. While the relevance and impact of ADO on the host cells as a potent immunosuppressive molecule is clear and accepted, its effects on cancer cells remain controversial. Depending on the specific ADO receptors expressed by tumor cells, both growth stimulation and inhibition were reported [22,32,44].

From now on, the attention of this review will be focused on CLL, dissecting expression, role and impact of the adenosinergic/purinergic axes in leukemic and bystander cells, finally discussing their relevance in a translational perspective.

## 3. Expression of the Adenosinergic Axis Machinery on CLL Cells

Similarly to solid tumor models, a role for ADO signaling in hematologic malignancies has also been described [45]. For example, in a human model of T cell leukemia, ADO promotes leukemic cell survival by inhibiting apoptosis induced by tumor necrosis factor-related apoptosis-inducing ligand (TRAIL) and contributes to the multidrug resistant phenotype of leukemic cells [46]. Over the past years, increasing evidence highlighted that deregulated surface expression of both ecto-enzymes and ADO receptors might carry negative prognostic relevance in cancers, including melanoma, breast and endometrial carcinomas, marking diseases with strong metastatic potential and immune tolerant environment [47,48]. This holds true also in the context of CLL, a disease characterized by an intense cross-talk between leukemic and non-tumor bystander cells in microenvironment-associated structures (e.g., lymph nodes (LNs) and bone marrow (BM)), where adenosinergic signaling is integrated in the complex network of cell-cell contacts and soluble mediators driving leukemia [49,50,51].

### 3.1. CD39 and CD73

CLL cells residing in the LN express high surface levels of both CD39 and CD73, although some variability across samples is reported [2]. CD39 was found to be homogeneously expressed at higher level in the Ki67^+^ CLL fraction compared to the resting population that showed heterogeneous but consistently weaker expression [52]. Overall, CD39 expression was reported to be consistently higher on circulating lymphocytes of CLL patients compared to healthy donors [53]. However, patient stratification according to clinical and molecular prognostic markers highlighted that CD39 expression on malignant B cells is generally associated with favorable prognosis. Indeed, CD39 was reported: (i) to inversely correlate with disease stage, being expressed at higher levels in stage 0–2 CLLs compared to stages 3–4 and (ii) with therapy requirement, being higher in treatment-naïve patients. In addition, expression of CD39 is enriched in samples carrying mutated *IGHV* genes, a common feature in good prognosis CLL subset [53,54]. Accordingly, CD39 ATPase and ADPase activities are enhanced in stage 0–2 CLL than in stage 3–4 [55]. The observation that expression of CD39 on CLL cells, despite being significantly higher than healthy subjects, represents a marker of good prognosis may sound contradictory. The explanation relies upon two considerations: (i) when looking selectively to the B cell compartment, CD39 expression and ATPase/ADPase activity were found to be quite similar in leukemic cells and B cells collected from healthy donors and (ii) in healthy people the T cell fraction represents the majority of circulating lymphocytes population, at variance with CLL patients. This prompted the idea of a major contribution of T lymphocytes in determining lower CD39 levels in normal samples [55]. Indeed, besides leukemic cells, CLL LNs are rich of stromal components that are intensely CD39^+^, including T lymphocytes [2]. The comparative analysis of T cell subsets highlighted that, overall, CLL patients have a higher percentage of CD39^+^ lymphocytes than controls, both in the CD4^+^ and in the CD8^+^ compartment. In contrast to what has been observed in malignant B cells, CD39 expression on T cells strongly associates with a more advanced disease stage. Consistently, CD39^+^ T lymphocytes are enriched in CLL patients that need to be treated, compared to untreated patients [56,57]. Ultimately, T-cell CD39 expression is considered predictive of treatment requirement, as a significantly shorter time to first treatment (TTFT) was observed in patients with CD39^high^ T cells compared to CD39^low^ [54].

In contrast to CD39, that is consistently present at high levels on CLL cell surface, expression of CD73 characterizes roughly 1/3 of CLL patients [2]. In the LNs, CD73 expression is significantly enhanced in the perivascular areas and in the proliferation centers, areas of intense leukemic cells proliferation with infiltration of CD4^+^/CD25^+^ T lymphocytes [58]. Accordingly, CD73 expression is higher in Ki67^+^ B CLL cells, which are in close contact with infiltrating T cells. When looking at the circulating fraction, the majority of samples expresses very low levels of CD73 on cell surface, with ≈30% CLL showing a clearly detectable CD19^+^/CD73^+^ population. CD73 expression associates with the co-occurrence of other negative prognostic markers such as CD38 and ZAP70, both functionally involved in the modulation of CLL homing to LN. In addition, CD73^high^ samples are enriched of a CD19^+^/Ki67^+^ population compared to the CD73^low^ counterparts. Altogether, these observations suggest that CD73 expression identifies a leukemic subset of cells characterized by: (i) an increased recirculation to and from the lymphoid niche; (ii) a more aggressive clinical behavior; and (iii) a higher cellular turnover [2]. Furthermore, it was proposed that higher CD73 expression may associate with time to disease progression after fludarabine treatment [59], thus corroborating the importance of this molecule in the CLL context.

CD39 and CD73 are also functionally active on CLL cell surface, and the metabolism of extracellular nucleotides/nucleosides can be efficiently monitored through reverse phase high-performance liquid chromatography (RP-HPLC), among other assays [60]. The amount of ADO resulting from sequential CD39^−^ and CD73^−^ mediated reactions is compatible with the micromolar range of concentrations reported for solid tumor tissues. It was observed that, despite virtually all CLL cells could degrade ADP to AMP as they constitutively express CD39, a certain proportion of cells expressing CD73 is needed to appreciate ADO production, with a cut-off of ≥30% positive cells. Furthermore, CD73^+^ cells consume significantly more ADP than the CD73^−^ counterparts, probably because of the lack of a feedback inhibitory mechanism controlled by AMP accumulation. Data obtained using specific inhibitors suggest that CD73 is the rate-limiting enzyme in the adenosinergic axis cascade. Therefore, ADO production in CLL is strongly dependent on CD73 expression, which is in turn tightly regulated by signals coming from the environment [2].

### 3.2. CD26 and Nucleoside Transporters

Extracellular ADO can be either converted to inosine and re-uptaken by the cell through concentrative or equilibrative nucleoside transporters (CNTs and ENTs, respectively) to reconstitute the intracellular pool [61], or it can bind P1 receptors. Deamination to inosine is mediated by ADA, an enzyme generally present in the cytoplasm but that can be localized on the cell surface when bound to anchoring proteins, such as CD26 [62]. CD26 plays a significant role in CLL biology, being both a disease marker and a modifier of tumor pathogenesis and development [63,64,65,66]. Several studies highlighted that expression of CD26 identifies CLL patients with high-risk of disease progression and treatment requirement [67]. Indeed, CD26 expression is strongly associated with other consolidated negative prognostic markers as CD49d [68], CD38, ZAP-70, and unmutated IGHV [69]. In addition to its integral membrane form, a soluble form of CD26 is detectable in the serum [70]. In the CLL field, the prognostic value of serum CD26 is limited, as they are not consistently increased in CLL patients compared to controls and do not correlate with other negative prognostic markers. Nevertheless, it was proposed that levels of soluble CD26 > 371 ng/mL might be of prognostic relevance in early disease (stage A), as they could identify patients with shorter TTFT [71].

CLL cells express four nucleoside transporters, namely hCNT2, hCNT3, hENT1, and hENT2, constitutively found at the mRNA level, whose protein expression is tightly modulated by different stimuli (e.g., phorbol 12-myristate 13-acetate, lipopolysaccharide, and tumor necrosis factor α), as well as nucleosides and nucleoside-derived drugs. The importance of nucleoside transporters in CLL stems from the fact that anti-leukemic therapy using nucleoside-derived analogs, such as fludarabine, depends on drug uptake and metabolic activation, and, therefore, the modulation of transporter expression on the cell surface may affect drug bio-availability [72]. Despite the apparent co-expression of these four transporter genes in CLL cells, most of the measurable natural nucleoside transport and all detectable fludarabine uptake into CLL cells rely on hENT-type carriers [73]. It was also documented that hENT2 protein expression significantly correlates with response of CLL cells to fludarabine ex vivo, potentially predicting therapeutic outcome in the clinical practice [74].

### 3.3. P2 and P1 Receptors

Among purinergic receptors, P2X7 is the most studied in cancer models [75,76] and several studies showed that it is the main ATP-activated channel in lymphoblastoid cells [77,78]. More recently, P2X7 over-expression has been suggested as a negative prognosticator in several malignancies, including prostate cancer [79], acute myeloid leukemia [80] and CLL [81,82].

Functional data on P2X7 in CLL are contradictory, with one paper suggesting that it may improve growth of CLL cells [82], while a second one highlighted its pro-apoptotic functions [83]. Adinolfi and colleagues reported that P2X7 expression and function are increased in CLL lymphocytes purified from patients with progressive disease, compared to patients with an indolent form of leukemia. The authors speculated that a higher expression of this receptor would result in a growth advantage for the leukemic clone, especially in unfavorable conditions, including limited availability of substrates or serum-derived growth factors [84]. They also showed that CLL cells exposed to high levels of exogenous extracellular ATP are more susceptible to its cytotoxic effect, through P2X7 activation and consequent stimulation of intracellular caspases, leading to apoptosis. Even if these results seem to be paradoxical, they are in line with what reported for other cellular models and can be explained by the diverse array of intracellular signals generated. In fact, under tonic low-level activation, P2X7 promotes survival and growth, but under massive stimulation, it triggers cell death [84,85].

P2X7 is the most polymorphic P2 receptor. Wiley and co-workers reported a single-nucleotide polymorphism (SNP) consisting of an adenine to cytosine substitution at position 1513 (1513A→C), resulting in the conversion of a glutamic acid to alanine at position 496 (E496A). This non-synonymous SNP is responsible for a loss of function of the P2X7, while not affecting its expression [86]. The SNP is also present at a greater frequency (three-fold increase) in a cohort of CLL patients characterized by an indolent disease compared to normal individuals [83]. Patients homozygous for the polymorphic allele show no P2X7 function, while heterozygous ones are characterized by an intermediate activity compared to wild-type homozygous individuals. The rate of ATP-induced apoptosis varies accordingly.

However, these results were not confirmed by subsequent studies. Rosenquist and colleagues reported that in a retrospective analysis in a cohort of 170 CLL patients and 200 healthy controls similar frequencies of the 1513C allele were found. Within the CLL cohort, the frequency of 1513C is independent of different prognosticators of the disease, including the immunoglobulin heavy chain (IGHV) mutational status [87]. Analysis on other large independent cohorts reached similar results with no correlation with age or stage at diagnosis, time to first treatment, progression-free survival and other prognostic markers [88,89,90]. Taken together, these results suggest that is unlikely that 1513C P2X7 polymorphism may have a significant role in the pathogenesis or progression of CLL.

Considering ADO receptors, only A2A is highly expressed by CLL cells, whereas levels of A1, A2B, and A3 are barely detectable and their role in CLL has not been yet addressed [2]. A2A is the receptor most commonly involved in anti-inflammatory and antitumor responses [91]. Compared to age- and sex-matched healthy donors, CLL patients show significantly higher levels of A2A and the expression of this receptor is further increased in response to signals inducing CLL proliferation, such as TLR9 agonists and IL-2. In addition, A2A levels in CLL cells do not show any evident correlation with CD73 surface expression, further corroborating the idea that CD73 represents the limiting factor in the adenosinergic axis activation in CLL [2].

## 4. ADO Signaling in CLL Cells

The A2A receptor on CLL cells is functionally active as, in response to a specific agonist (CGS21680), intracellular cAMP concentrations increase because of the activation of the receptor-coupled stimulatory G proteins. Exposure of CLL cells to TLR9 agonists and IL-2 further enhances cAMP release in response to CGS21680, confirming that in activated CLLs the adenosinergic axis is boosted [2].

Signaling downstream of ADO receptors is a fundamental component of the immunosuppressing machinery having regulatory T cells as main players and blunting T cell proliferation and secretion of T-helper 1 (Th1) cytokines [92]. However, less characterized are the effects that the adenosinergic axis exerts through autocrine mechanisms on leukemic cells, effects that impact on critical features of CLL biology, such as homing and survival.

### 4.1. ADO Signaling Modulates CLL Cells Homing

The finding that CD73 expression in CLL LNs is higher on cells residing in perivascular areas, along with its association with CD38 and ZAP70, both markers of cells with high recirculating ability, suggested that the adenosinergic axis might be relevant in the homing process and in tuning chemotactic responses in CLL. Several pieces of evidence in literature highlighted that adenosinergic signaling modulates the migration of both tumor and immune cells [93,94]. In other tumor models, A2A effects are believed to be due to the down-modulation of chemokine receptors on cell surface [95], or on receptor desensitization by cAMP [96]. In line with these observations, CD73-produced ADO inhibits CLL cell migration toward C-X-C motif chemokine 12 (CXCL12), one of the major drivers of lymphocytes recirculation to and from LNs [97]. The proposed mechanism behind this effect relies upon ADO-mediated down-modulation of chemokine receptors, as intensity of C-X-C chemokine receptor type 4 (CXCR4) levels expressed on CLL is significantly decreased in the CD73^+^ subset compared to CD73^−^ [2]. The inference would be that leukemic B cells are attracted to LNs because of the long-range signal provided by CXCL12 and other chemokine gradients, whereas local ADO triggers a short-range stop signal capable of keeping cells in a growth-supportive environment.

### 4.2. ADO Signaling Rescues CLL Cells from Spontaneous- or Drug-Induced Apoptosis

One of the major contributions of LN microenvironment to CLL is to provide survival signals to leukemic cells and to protect them from spontaneous and drug-induced apoptosis, thereby maintaining a leukemic cell reservoir that fuels disease progression [13]. The role of ADO on apoptosis is controversial, as both pro-apoptotic [98] and cyto-protective [99] effects have been reported. One possible explanation relies on the expression of different ADO receptors capable of inducing or blocking the synthesis of intracellular cAMP thereby modulating its concentrations. In the CLL field, Serra and co-authors demonstrated that extracellular ADO protects cells both from spontaneous and drug-induced apoptosis, in a range of concentrations compatible with that of CD73-mediated ADO generation by CLL cells. ADO exerts a dose-dependent protective effect counteracting or limiting the efficacy of different chemotherapeutic agents independently of their mechanism of action. For example, in vitro, ADO significantly impairs the effects of etoposide, a DNA-damaging drug that rapidly triggers robust apoptosis in CLL. This effect can be recapitulated by the selective A2A agonist CGS21680, and is completely lost if cells are pretreated with the A2A inhibitor SCH58261, indicating that ADO-mediated protection from apoptosis is mainly acting through the A2A receptor. In line with this observation, inhibition of CD73 resulted in a small decrease in cell viability when used as a single agent, but acted synergistically with etoposide strongly potentiating the apoptotic effect of chemotherapy. Addition of exogenous ADO rescues cells from apoptosis confirming the key role of CD73 in fueling CLL cells with ADO. From a functional standpoint, exposure of leukemic cells to ADO in combination with etoposide results in the modulation of the expression of p53-dependent elements, including Mcl-1 and BAX, finally resulting in a decreased activation of caspase-3 [2]. These observations are consistent with results highlighting a protective effects induced by intracellular cAMP through the activation of the NF-κB pathway, which in turn impairs the apoptotic machinery triggered by p53 [100,101].

Similar effects were observed when exposing CLL cells to fludarabine, where ADO conferred complete protection to CLL cells, although the effect is not fully recapitulated by the A2A agonist, opening to the possibility that in this context ADO should act through both A2A-dependent and -independent mechanisms [2]. The explanation may rely upon the remarkable similarity in the structure of ADO and fludarabine, likely competing for nucleoside transporters [59] and for intracellular effectors. According to this hypothesis, in the presence of high amount of extracellular ADO, fludarabine would have a limited access to its final targets, in line with one of the suggested mechanisms behind fludarabine resistance in leukemia [72].

## 5. ADO in the CLL Niche Is Part of a Network of Micro-Environmental Signals

Recent data shows that the CLL niche within the LN is a highly hypoxic microenvironment where low oxygen tension activates a specific genetic program mainly through the regulation of HIF1α [102]. HIF1α modulates the expression of several molecules involved in the leukemic niche dynamics, including chemokine receptors and cell adhesion molecules, such as CXCR4 and VLA-4 respectively, suggesting that it plays a role in controlling the cross-talk of leukemic cells with BM and spleen microenvironments. Remarkably, *HIF1α* mRNA levels in circulating CLL vary significantly within patients, and HIF1α is upregulated, at the transcriptional level, in leukemic cells upon contact with stromal cells in a positive feedback loop that may foster CLL expansion and protection [103]. For this reason, it was recently proposed that *HIF1*α mRNA overexpression in circulating B cells might be considered a promising independent molecular marker for adverse prognosis CLL [104]. When comparing HIF1α expression in leukemic cells collected from paired peripheral blood (PB), BM, and LNs of CLL patients, mRNA levels are the highest in LN, together with known HIF1α target genes. Accordingly, immunohistochemical analyses of HIF1α and its target carbonic anhydrase IX (CAIX) indicate the presence of a hypoxic gradient in CLL LN with an inverse correlation between the intensity of CAIX staining and the distance from vessel, and highlight the proliferation centers as the areas with the lowest oxygen tension [3].

**Figure 2:**
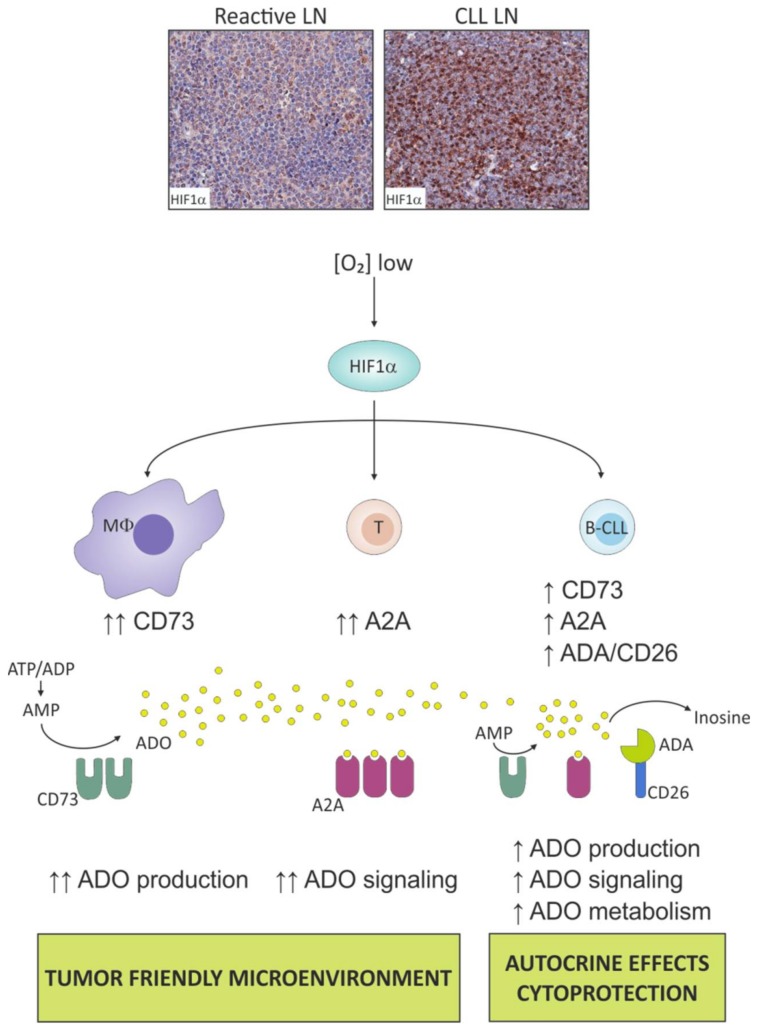
ADO in the chronic lymphocytic leukemia (CLL) lymphoid niche is part of a complex network of micro-environmental signals. The CLL niche is a hypoxic environment, as witnessed by high levels of HIF1α compared to reactive lymph nodes (LN) from healthy individuals. HIF1α modulates the adenosinergic axis both in bystander and CLL cells. These populations expressed on their cell surface the enzymatic machinery to dismantle ATP/ADP/AMP, generating ADO. This nucleoside can exert paracrine or autocrine effects, mediated at least in part through the binding to A2A receptor, or can be dismantled to inosine.

Several pieces of evidence highlighted that hypoxia acts through ADO signaling in inflammation and cancer [105,106,107,108] and specifically activating, at least in part, the A2A receptor signaling. It was proposed that tumor protection from CD4^+^ and CD8^+^ T cells is due to a large extent to the inhibition of antitumor T cells by hypoxia-driven local accumulation of extracellular ADO in the microenvironment, resulting from tumor metabolic adaptation to low oxygen tension [109]. A reciprocal cross-talk between hypoxia and adenosinergic axis has been reported with HIF1α directly regulating the expression of elements of ADO signaling [110]. This holds true also for CLL where the overexpression and activation of HIF1α in the LN increases ADO generation and signaling, affecting tumor and host cellular responses (Figure 2).

### 5.1. Hypoxia Boosts the Adenosinergic Axis in CLL Cells

Experimental evidence shows that CD73 expression is significantly upregulated under in vitro hypoxic conditions, both at the mRNA level (*NT5E*) and on CLL cell surface [3], in line with previous observation of a direct transcriptional control of *NT5E* by HIF1α [111]. This effect is restricted to the CLL subset with >30% CD19^+^/CD73^+^ cells as no significant modulation is observed in the negative subset. These data, along with the fact that CD39 expression levels remain unmodified upon oxygen tension variation, further consolidate the pivotal role of CD73 in the adenosinergic axis. All the sequential steps of the AMP degradation chain downstream of CD73 are markedly upregulated in response to hypoxia, as indicated by significant increases in the expression of CD26 together with nucleoside transporters (ENT1 and ENT2) and A2A receptor. This expression profile suggests that under hypoxic conditions, CLL cells upregulated the enzymatic machinery for ATP scavenging with rapid degradation of ATP to inosine and subsequent cellular uptake. Consistently, CLL samples collected from LN show a strong upregulation of CD73, CD26, ENT1-2, and A2A, which are expressed at significantly higher levels compared to paired PB samples, fostering the idea of a strong ADO signaling in the LN compartment [3].

### 5.2. Hypoxia Fosters the Adenosinergic Axis in “Not So Innocent” Bystander Cells of the Leukemic Niche

When CLL cells are cultured under hypoxic conditions, they do not accumulate ADO in the extracellular space, likely because this nucleoside is rapidly converted to inosine, as the adenosine deaminase activity is also upregulated under hypoxia. This observation suggests that the hypoxia-ADO circuit is not merely restricted to leukemic cells through an autocrine self-fostering mechanism, but involves other elements of the LN environment in a more complex network of interactions. Indeed, hypoxia induces a strong upregulation of the adenosinergic axis also in nurse-like cells (NLCs), a type 2 (M2) macrophage population residing in the LN that secretes cytokines supporting tumor growth and suppressing immune response [112]. When derived in vitro and exposed to low oxygen tension, NLCs significantly upregulated CD73 levels and ADO production, thereby fueling A2A signaling of surrounding cells as well as activating autocrine signaling through MAPK, PI3K, and NF-κB [3]. Indeed, expression of A2A is also increased in NLCs upon hypoxia and signaling through ADO receptor further contributes to M2 macrophage polarization, as revealed by gene expression profiling. Hypoxia upregulates, among others, the expression of the transcription factor IRF4 and of the tryptophan-metabolizing enzyme indoleamine 2,3-dioxygenase (IDO), both M2 macrophages markers [113], and this effect is prevented by A2A antagonist. Furthermore, the hypoxia-ADO signaling strengthens the growth-supportive ability of NLCs by increasing synthesis of IL-6, which confers growth advantage to CLL cells. Therefore, the major inference of ADO signaling activation in the CLL microenvironment is the re-shaping of a tumor-favorable niche with tumor-supportive and immunosuppressive features. This holds true also when looking at the T cell population. ADO mediates the hypoxia-induced impairment of T cell proliferation in response to activatory stimuli, such as anti-CD3/CD28 ligation, and promotes the development and expansion of a T cell population with regulatory phenotype and suppressive properties (Tregs).

### 5.3. Hypoxia Contributes to Metabolic Skewing of Cells in the Leukemic Niche

CLL T lymphocytes cultured under hypoxic conditions switch their metabolism toward glycolysis, with robust upregulation of glucose and lactate transporters and of lactate dehydrogenase (LDHA) and pyruvate kinase 2 (PKM2) [3]. In line with the observation that metabolic switch toward glycolysis skews T cells functions to a regulatory phenotype [114], CLL T lymphocytes increase the expression of FOXP3 together with PD-1, IL-10, and VEGFA. In contrast, mRNA levels of interferon γ (*IFNG*) are markedly reduced, and these effects can be recapitulated upon pharmacological modulation of A2A activation [3].

**Figure 3:**
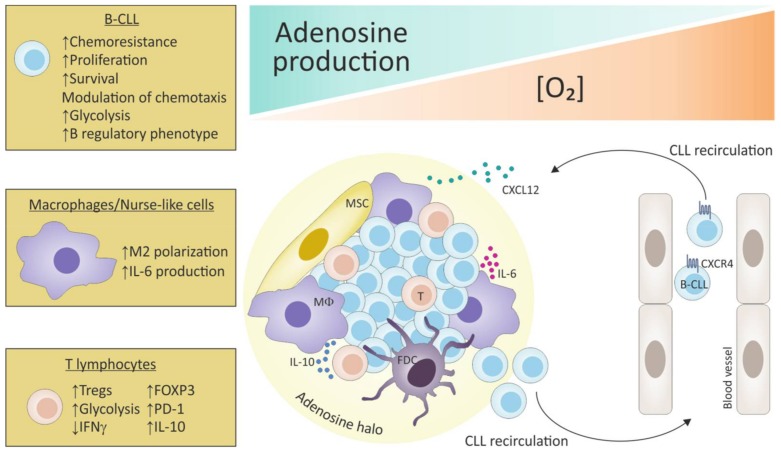
Adenosine-mediated immunosuppression in the CLL lymphoid niche. CLL cells can recirculate from the periphery to lymphoid organs following chemokine gradients, including CXCL12. Once in the tumor niche, leukemic cells are in closed contact with macrophages (MΦ)/nurse-like cells, mesenchymal cells (MSC), follicular dendritic cells (FDC) and T lymphocytes. These areas are characterized by low oxygen tension and high concentrations of ADO, which forms a halo able to protect CLL cells from the effects of chemotherapy, while increasing proliferation, survival and shifting their metabolism towards a glycolytic one. At the same time, ADO skews T cells and macrophages towards an immune-tolerant and CLL-supportive phenotype, mediated also through the secretion of specific cytokines (IL-6/IL-10).

Metabolic adaptation upon oxygen deprivation is a common feature of tumor cells that rapidly foster energy production via glycolysis through HIF1α-mediated transcriptional control [115]. Accordingly, at 1% O_2_ leukemic B cells markedly increase glycolysis as evaluated both through the dynamic monitoring of metabolic responses and the expression of glycolysis genes such as those encoding the glucose transporter GLUT1 (*SLC2A1*), *LDHA*, the lactate transporter monocarboxylate transporter 4 MCT4 (*SLC16A3*), and *PKM2*. Once again, this effect is partly mediated through the activation of ADO signaling, as indicated by the complete reversion of the glycolytic behavior of CLL cells upon the addition of A2A inhibitor, directly linking ADO signaling to central metabolic programs. This prompts the idea that leukemic cells in the areas of intense proliferation undergo a hypoxia-driven ADO-mediated metabolic adaptation with a shift towards glycolysis for the correct energy supply [116]. In keeping with the immunomodulatory effects registered in the T cell compartment, the pattern of metabolic changes induced by the hypoxia-ADO axis on leukemic B cells also plays an important role in creating local conditions of immunosuppression by tuning cytokine production and release. CLL cells cultured under hypoxic conditions acquire a B-regulatory phenotype as defined by a marked upregulation of IL-10 [117], that is prevented upon A2A antagonist, thus confirming that in CLL microenvironment hypoxia and ADO function in a common signaling axis [3] (Figure 3).

### 5.4. Targeting the Adenosinergic Pathway

As highlighted in this review, in CLL, as well as in several cancer models, the activity of ATP and its derived metabolites is critical for cancer growth and polarization of the immune system toward a tumor-supportive condition. Thus, it is reasonable to hypothesize to target purinergic/adenosinergic signaling in the fight against cancer. This could result in direct effects on cancer cells through the reduction of the ADO levels present in the tumor microenvironment as well as in the re-activation of the host response by the maintenance of increased levels of ATP able to activate purinergic receptors and to recruit immune cells into the tumor.

This paragraph will explore how these pathways have been targeted in several tumor models showing that ADO scavenging prevents tumor progression and metastasis. These different approaches are presented here because they could be applied to CLL, where at the moment only very few preliminary data are available. Distinct levels of targeting can be suggested in the attempt to lower extracellular concentrations of this nucleoside and modulate its effects. First, a decrease of ADO can be obtained through the downregulation of CD39 and/or CD73 [118,119,120] or through the upregulation of CD26 or ADA. Several CD39 inhibitors, including ARL67156 and POM-1, have shown efficacy in animal models of follicular lymphoma, sarcoma, or murine melanoma, resulting in a partial overcome of T-cell hypo-responsiveness to stimulation [121], increased therapeutic response to chemotherapeutic agents [122], or inhibition of tumor growth [123], respectively. Even the use of specific anti-CD39 antibodies able to interfere with the enzymatic activity of the molecule seems to be effective in the tumor context, as recently reported in sarcoma and ovarian cancers [124,125,126]. The promising value of targeting the CD39-CD73-ADO cascade as an anti-tumor therapy has been further demonstrated by treatment of mice with an anti-CD73 antibody that curbs the development of lung metastases in a breast tumor model [120]. Similar results were obtained by other groups [118,119,127,128,129,130]. Highly promising in a translational perspective is the compound a,b-methylene-ADP (APCP), the most potent competitive CD73 inhibitor [45,131,132]. A therapeutic anti-CD73 antibody, MEDI9447 is currently in Phase 1 clinical trial in cancer patients (NCT02503774) [133,134].

A second level of targeting is represented by the blockade of the A2A/A2B ADO receptors or the activation of the A3. Several studies have shown that administration of A2A antagonists can enhance anti-tumor immunity in preclinical models [135,136,137]. Moreover, targeting both tumor and host A2B has been reported to decrease tumor growth and metastasis, promoting anti-tumor immunity [47,138,139,140]. At least three different A2A antagonists are in Phase I clinical trials (CPI-444, PBF-509, and AZD4635), both alone and in combination with anti-PD1/PD-L1 therapy for solid tumors. Recently, a dual A2A/A2B inhibitor, AB928, has been designed to overcome potential compensatory mechanisms due to the inhibition of one of these two receptors [141]. AB928 is in phase I trial in healthy volunteers and expect to initiate a phase 1/2 trial in cancer patients in the first half of 2018.

A third level of control and targeting of the adenosinergic signaling is the downregulation of ADO receptor. In adoptive immunotherapy, anti-tumor effector cells may be manipulated to be irresponsive to ADO by silencing receptor expression with siRNA or by exposing the cells to ADO receptor agonist. These treatments result in the abrogation of ADO-mediated immunosuppression in tumor microenvironment, promoting, at the same time, eradication of tumor cells by immune system. Otha and colleagues showed that silencing of A2A in T cells inhibited tumor growth and metastasizing while preventing neo-vascularization [23,142].

Given the promising results obtained by a single-agent targeting, combination strategies can synergistically enhance anti-tumor immune responses. As immune cells infiltrating the tumor co-express CD39 in association with other co-inhibitory molecules (e.g., CTLA4 and PD-L1), a combinational approach targeting both CD39, CD73, and co-inhibitory molecules has been proposed. The aim is to control the immunosuppressive potential of ADO signaling while minimizing the side effects of anti-CTLA4 and anti-PD1 blockade [124,130,143,144,145]. Inhibition of ADO signaling has been shown to synergize with anti-PD-1 or anti-CTLA-4 mAbs in preclinical studies [129,135,137]. Phase 1 clinical trials evaluating CD73 or A2A blockade in combination with PD-1/PD-L1 inhibitors are currently being conducted (NCT02503774 and NCT02655822). Targeting A2A or CD73 in combination with adoptive cell therapy is another promising combination [45]. Accumulating evidence suggests that chemotherapy [146] and radiotherapy [147] can also synergize with immunotherapies. Consistent with this notion, inhibition of CD73, A2A, or A2B has been shown to enhance the activity of chemotherapy [47,127,139].

What is the state-of-the-art of targeting the adenosinergic axis in CLL? There are only few papers addressing this point. In a study published in 2005, Balakrishnan and co-workers demonstrated that leukemic lymphocytes treated with 8-Cloro-Adenosine (8-Cl-Ado) were characterized by a time- and dose-dependent increase of 8-Cl-ATP, with a concomitant decrease of the endogenous ATP pool. Inhibition of global RNA synthesis resulted in a significant decline in the expression of transcripts involved in the apoptotic pathway, including Myeloid cell leukemia-1 (MCL1), a key survival factor for CLL cells. Furthermore, 8-Cl-ATP resulted in programmed cell death, as inferred by multiple read-outs including caspases activation, cleavage of caspase-3, and PARP (poly-adenosine diphosphate [ADP]-ribose polymerase), and enhanced DNA fragmentation. These results indicated that 8-Cl-Ado is able to induce apoptosis in CLL lymphocytes by targeting cellular bioenergy as well as RNA transcription and translation of key survival genes such as MCL1 [148]. This nucleoside analogue has entered a phase I-II clinical trial for patients with CLL (NCT00714103) and acute myeloid leukemia (AML) (NCT02509546) and Stellrecht and colleagues showed that it acts by inducing autophagy in CLL cells both in vitro and in vivo during therapy [149].

### 5.5. Future Perspectives

The increasing literature highlighting the clinical benefits of targeting the adenosinergic pathway in cancer, together with in vitro data underlying its functional role in CLL, open the way to novel additional therapeutic strategies needed as none of the current treatment options for CLL are ultimately disease curative.

To this purpose, the Eμ-TCL1 mouse model [150], based on the adoptive transfer of spleen-purified leukemic cells in naïve C57BL/6 immunocompetent recipients, represents a useful tool to study CLL and for preclinical analyses. Concerning the adenosinergic axis, data so far obtained indicate that CD39 is expressed at very high levels by TCL1 leukemic cells compared to the normal B cell counterpart from naïve mice. Similar to CLL patients, high heterogeneity in CD73 expression is observed, supporting the idea of variable levels of ADO in tissue colonized by leukemic cells. Moreover, leukemic cells are characterized by low levels of CD26 in contrast to wild-type B cells [151].

Specific T cell and myeloid subset changes are associated with CLL development. Indeed, TCL1 mice are characterized by a drop of the naïve T cell subset compared to WT mice, with simultaneous over-representation of memory, exhausted and regulatory T cell sub-populations, in line with published data [152,153]. Similarly, the monocyte compartment undergoes major shifting towards an M2 tumor-supportive phenotype, with the patrolling population overcoming the inflammatory one, consistent with data reported by Hanna and colleagues [154]. Taken together, these results show that the adenosinergic axis is over-expressed in the TCL1 mouse model and that the presence of leukemic cells polarizes the host immune system, favoring the establishment of a tumor supportive environment [151]. These observations confirm what described for CLL patients and pave the way to test the impact of ADO-targeting therapies, alone or in combination with drugs that directly target the leukemic clone to magnify therapeutic responses.

## 6. Concluding Remarks

The emerging picture in cancer therapy is that better results in terms of clinical responses can be achieved by a “double hit” strategy, targeting tumor cells and tumor-host interactions. For these reasons, a deep characterization of all the mechanisms contributing to cancer development, progression, and immune-escape is an essential step. Nucleotides, nucleosides, and their receptors are part of this network and they play a critical role in contributing to cancer cells growth and in shaping the tumor microenvironment through different circuits.

In vitro and in vivo results from several tumor models strongly indicate that altering the balance between ATP and ADO, thus interfering with the adenosinergic axis, has significant antitumor effects. This approach could represent a winning strategy also for CLL. CLL is a hematological malignancy strongly dependent on tumor microenvironment interactions to derive growth signals as well as protection from drugs and immune system. In this context, the leukemic niche is a safe environment, contributing to the maintenance of disease reservoir. CLL niche is a hypoxic environment that fosters the adenosinergic axis by generating a tumor-protective halo of ADO, through the modulation of the enzymatic machinery able to dismantle ATP and the receptors capable of using ADO. The final results are cytoprotection of leukemic cells from chemotherapy and modulation of immune system responses through the polarization of T and myeloid cells towards a pro-tumorigenic phenotype. Thus, it is reasonable to hypothesize that in CLL the adenosinergic axis may be a good candidate for targeting acting at two different levels: on one side, by preventing the generation of an ADO-protecting halo and on the other side by awaking the immune system against the leukemic cells.

The whole picture that emerges from this increasing body of results paves the way to further investigate on the adenosinergic/purinergic signaling in CLL and on the potential impact of interfering compounds, opening promising perspective to develop novel therapeutic strategies.
